# Metal resistant PGPR lowered Cd uptake and expression of metal transporter genes with improved growth and photosynthetic pigments in *Lycopersicon esculentum* under metal toxicity

**DOI:** 10.1038/s41598-019-41899-3

**Published:** 2019-04-10

**Authors:** Kanika Khanna, Vijay Lakshmi Jamwal, Sumit G. Gandhi, Puja Ohri, Renu Bhardwaj

**Affiliations:** 1 Department of Botanical and Environmental Sciences, Guru Nanak Dev University, Amritsar, 143005 India; 2 Indian Institute of Integrative Medicine (CSIR-IIIM), Council of Scientific and Industrial Research, Canal Road, Jammu, 180 001 India; 3 Department of Zoology, Guru Nanak Dev University, Amritsar, 143005 India

## Abstract

Plant growth promoting rhizobacteria (PGPRs) are very effective in immobilization of heavy metals and reducing their translocation in plants via precipitation, complex formation and adsorption. The present study was therefore designed to understand the role of *Pseudomonas aeruginosa* and *Burkholderia gladioli* in mitigation of Cd stress (0.4 mM) in 10-days old *L*. *esculentum* seedlings. The present work investigated growth characteristics, photosynthetic pigments, metal tolerance index, metal uptake and the contents of metal chelating compounds (protein bound and non-protein bound thiols, total thiols) in microbes inoculated Cd treated *L*. *esculentum* seedlings. The gene expression profiling of different metal transporters was conducted in order to investigate the quantitative analysis. Our results revealed Cd generated toxicity in seedlings in terms of reduced growth (root length, shoot length and fresh weight) and photosynthetic pigments (chlorophyll, carotenoid and xanthophyll) which enhanced upon inoculations of *P*. *aeruginosa* and *B*. *gladioli*. Further, the metal uptake along with levels of protein and non-protein bound thiols was also enhanced in Cd-treated seedlings. Gene expression studies suggested enhanced expression in the metal transporter genes which were further declined in the microbe supplemented seedlings. Therefore, micro-organisms possess growth promoting traits that enable them to reduce metal toxicity in plants.

## Introduction

Heavy metals have been widely distributed and a prominent apprehension for sustainable agriculture and human welfare^1,2^. They have been discharged in the environment through anthropogenic activities such as fertilizers, industrial wastes, pesticides, mining, sewage etc.^3^. Many of these metals act as vital nutrients for plant growth under optimal concentrations but when they exceed their optimal limits, they tend to be toxic in nature^4^. There is undoubtedly an urgent need to promote crop productivities by eliminating the metal toxicities from food chain^5^.

Cd is highly toxic and non-essential heavy metal existing in the environment that leads to reduction in the plant productivities^6^. It shows higher affinity for sulphur in acidic conditions because of its mobile nature, thereby promoting absorption towards roots^6^. The translocation of heavy metals in plants occurs through sympast or apoplast or with the help of different transporters^7^. It causes biochemical and physiochemical disturbances within plants by inhibiting their growth, photosynthesis, pigments, nutrient uptake, germination, electron transport chain, etc.^8^. Many vegetable crops can lead to the accumulation of Cd, causing severe damages towards crop productivity^9^. To maintain the quality and standards of vegetable crops there is an urgent need for remediation of these metal polluted soils. Different physio-chemical and biological methods of remediation have been employed in order to remove heavy metal pollutants from the fields^10^. The most commonly used technique for heavy metal remediation is *in situ* immobilization of metals through supplementation of different inorganic and organic agents such as phosphate salts, biochar, zeolites, manure, lime etc. This technique is known to be highly efficient, practically applicable and simple along with the economic benefits^11,12^. It has also been observed that these amendments can elevate pH levels and decrease the bioavailability of metals to plants at the polluted sites^13^. They have also been reported to prevent the translocation of heavy metals to food chain via precipitation, complex formation and adsorbing properties^10^, but many metal immobilizers are toxic for the sound quality of water and soil^14^. Therefore, there is the need for metal immobilizers which can remediate the multi-metal contaminated sites in eco-friendly manner along with the restoration of the soil properties and plant health^15,16^.

Metal immobilising and metal-resistant plant growth promoting rhizobacteria (PGPR) have recently been studied to stimulate growth and reduce the bioavailability of heavy metals and their accumulation within plants^10,17–19^. It was reported that *Bacillus megaterium* promoted growth and decreased uptake and translocation of Ni in *Brassica juncea*, *Luffa cylindrica* and *Sorghum halepense* plants^20^. It was demonstrated that *Neorhizobium huautlense* T1–17 stimulated the radish and Chinese cabbage growth under Cd and Pb stress and reduced their accumulation within the plant tissues^21^. Moreover, metal resistant and growth promoting bacteria provides resistance to plants in metal polluted sites by production of plant hormones such as indole-3-acetic acid (IAA), gibberellins etc. They also lead to the production of solubilising phosphates, siderophores and 1-aminocyclopropane-1-carboxylate (ACC) - deaminase that promote the plant growth and defense properties and reduce the translocation of heavy metals within the plant tissues^20,22,23^.

It has been reported that Cd pollution in different plant species is linked to Fe deficiency due to their alike chemical characteristics^24^. Normally, the lower Fe concentration in different plant species raised under Cd contamination lead to chlorosis due to inhibition of their chloroplast and chlorophyll synthesis^25^. The microbes tend to bind Fe and form siderophore complexes so as to provide Fe to plants. In addition to this, plant growth promoting bacteria can also bind directly with these heavy metals in order to reduce their bioavailability and toxicity^26^. Furthermore, micro-organisms also result in the upregulation of plant defense system, heavy metal tolerant proteins (MTP family) and phytohormones under Cd stress^27^. The micro-organisms also result in the regulation of metal transporter genes (*ZIP* gene family) upon Cd exposure that further reduces its accumulation in *Zea mays*^21^. *Bacillus subtilis* is well known metal resistant bacteria that was reported to reduce the Cd accumulation and promote plant growth in *Oryza sativa* grown under Cd stressed conditions^27,28^. It was further suggested by Guo *et al*. that *Burkholderia* sp. D54 significantly promoted the growth in *Sedum alfredii* by oxidising Fe(II) and Mn (II) that further forms Fe precipitates and arrest the uptake of metals upon root surfaces^29^. Similar trends were also found by Dong *et al*. in which inoculation of *Burkholderia* sp. decreased Cd translocation in rice and elevating the photosynthetic efficiencies in plants^30^. A better interpretation of characteristics of metal-resistant and metal-immobilizer plant growth promoting bacteria is a crucial prerequisite for establishment of effective and eco-friendly strategies for crop production in metal polluted environment.

*L*. *esculentum* is the most important, widely consumed crop and is quite sensitive to heavy metal stress. It is rich in nutrients and contains vitamins, lycopene, antioxidants and essential minerals^31^. Cd has severely affected the yield of tomato plants worldwide. The diversity of micro-organisms present in the rhizosphere shows mutualistic interactions and positively regulate the growth and metabolism of the plant under normal as well as stressed conditions. They are well known to ameliorate Cd toxicities by immobilization and reducing their translocation to the plant tissues.

The objectives of the present study were to explore the role of *Pseudomonas aeruginosa* and *Burkholderia gladioli* in 10-days old *L*. *esculentum* seedlings grown under Cd stress (0.4 mM). The effects of these strains were observed upon growth characteristics, photosynthetic efficiencies, metal tolerance index and metal uptake. Also the contents of metal chelating compounds such as protein bound thiols, non-protein bound thiols ant total thiols were assessed. The role of different metal transporters involved in Cd uptake and translocation were also analysed through expression profiling using qRT-PCR.

## Results

The significant observations on growth, photosynthetic pigments, metal tolerance index and its uptake, metal chelating compounds and expression profiling of the metal transporters are presented as below:

### Growth parameters

The effect of micro- organisms (*P*. *aeruginosa* (M1) and *B*. *gladioli* (M2)) on growth parameters of 10-day old *L*. *esculentum* seedlings under Cd stress was assessed in terms of root length, shoot length and fresh weight of the seedlings. It was observed that Cd stress resulted in reduction of the growth attributes of seedlings. Cd stress declined the root length and shoot length of seedlings by 32 and 31.61% in comparison to control plants. Furthermore, 35.64% decrease in the fresh was observed respectively. But supplementation of *P*. *aeruginosa* (M1) enhanced the root length, shoot length and fresh weight by 41, 39.8, 36.8%. Also, the application of *B*. *gladioli* (M2) improved the root length, shoot length and fresh weight by 48.2, 48.4, 52.7% respectively as compared to plants under Cd stress. Two-way ANOVA revealed the significant differences between root and shoot lengths and fresh weight (Table 1).

**Table 1:** Effect of M1 (10^9^ cells/ml) and M2 (10^9^ cells/ml) on growth parameters.

Treatments	Root length (cm) (Mean ± SD)	Shoot Length (cm) (Mean ± SD)	Fresh weight (mg) (Mean ± SD)
CN	7.10 ± 0.230^b^	5.91 ± 0.094^c,d^	10.28 ± 0.168^c^
Cd	4.78 ± 0.355^d^	4.04 ± 0.180^e^	7.58 ± 0.275^d^
M1	8.48 ± 0.278^a^	6.95 ± 0.075^a^	13.18 ± 0.382^a^
M1 + Cd	6.77 ± 0.220^b,c^	5.66 ± 0.38^c,d^	10.38 ± 0.248^c^
M2	7.42 ± 0.404^b^	7.21 ± 0.100^a^	12.65 ± 0.220^a^
M2 + Cd	7.09 ± 0.190^b,c^	6.00 ± 0.123^b,c^	11.58 ± 0.238^b^
F- ratio_(df 1,12)_ T	112.65**	265.103**	310.74**
F- ratio_(df 2,12)_ D	55.81**	124.805**	264.014**
F- ratio_(df 2,12)_ T × D	18.411**	5.340*	20.411**
HSD	0.795	0.521	0.722

^a^Root length. ^b^Shoot length and ^c^Fresh weight in 10-days old *L*. *esculentum* seedlings under Cd stress (0.4 mM). Data is presented as means of 3 replicates ± S.D (standard deviation) and HSD values. F ratio values, * indicates significance at P ≤ 0.05 and ** indicates significance at P ≤ 0.01). Different letters on the graphs indicate that mean values of treatments are significantly different at p < 0.5 according to Tukey’s multiple comparison test (CN-Control, Cd-Cadmium, M1-*Pseudomonas aeruginosa*, M2- *Burkholderia gladioli*).

### Photosynthetic pigments

**Figure 1:**
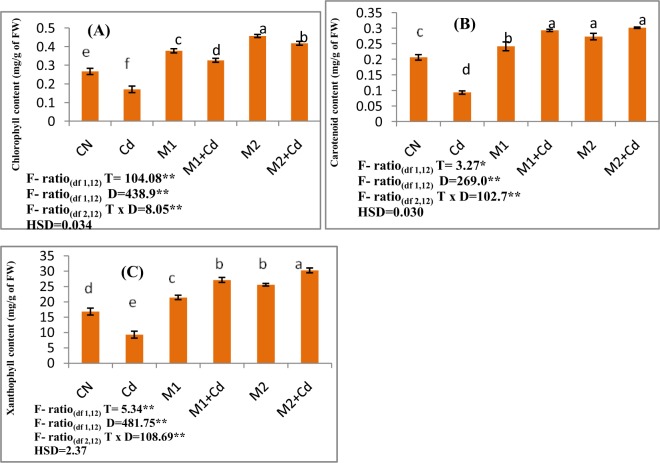
Effect of M1 (10^9^ cells/ml) and M2 (10^9^ cells/ml) on photosynthetic pigments. (**A**) Chlorophyll content (**B**) Carotenoid content (**C**) Xanthophyll content in 10-days old *L*. *esculentum* seedlings under Cd stress. Data is presented as means of 3 replicates ± S.D (standard deviation) and HSD values. F ratio values, *indicates significance at P ≤ 0.05 and **indicates significance at P ≤ 0.01). Different letters on the graphs indicate that mean values of treatments are significantly different at p < 0.5 according to Tukey’s multiple comparison test (CN- Control, Cd-Cadmium, M1- *Pseudomonas aeruginosa*, M2- *Burkholderia gladioli*).

The photosynthetic pigments were determined by analysing total chlorophyll, carotenoid and xanthophyll contents. Total chlorophyll content was lowered by 35.9% with 0.4 mM Cd concentration as compared to control seedlings. Seedlings treated with *P*. *aeruginosa* (M1) resulted in increased total chlorophyll content by 91.05%. Application of seedlings with *B*. *gladioli* (M2) also elevated the total chlorophyll content of the seedlings grown under Cd stress by 144.7%. The carotenoid content decreased by 54.7% in 0.4 mM Cd treated seedlings relative to untreated seedlings. Inoculation of *P*. *aeruginosa* (M1) led to elevation in carotenoid content by 213.6% in 0.4 mM Cd treated seedlings whereas seedlings amended with *B*. *gladioli* (M2) by 222.7% respectively. Further, xanthophyll content was observed to be declined by 44.8% in Cd stressed seedlings. An increase in the contents of xanthophylls by 192.3 and 225.8% was noticed after augmentation of *P*. *aeruginosa* (M1) and *B*. *gladioli* (M2) in Cd stressed seedlings (Fig. 1).

### Heavy metal tolerance Index and accumulation

Heavy metal tolerance index was observed to be lowered from 100% in control seedlings to 40% in 0.4 mM Cd stressed plants. An elevation in the tolerance index was recorded by 51.1 and 76% when Cd stressed seedlings were amended with (*P*. *aeruginosa* (M1) and *B*. *gladioli* (M2) respectively.

**Figure 2:**
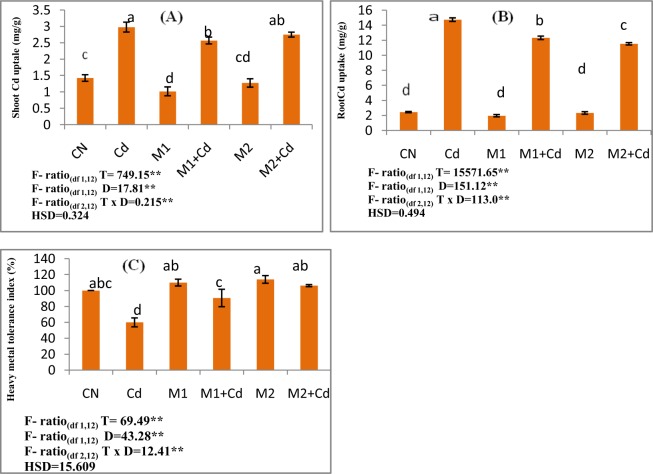
Effect of M1 (10^9^ cells/ml) and M2 (10^9^ cells/ml) on (**A**) Shoot metal uptake (**B**) Root metal uptake (**C**) Heavy metal tolerance index in 10-days old *L*. *esculentum* seedlings under Cd stress. Data is presented as means of 3 replicates ± S.D (standard deviation) and HSD values. F ratio values, * indicates significance at P ≤ 0.05 and ** indicates significance at P ≤ 0.01). Different letters on the graphs indicate that mean values of treatments are significantly different at p < 0.5 according to Tukey’s multiple comparison test (CN-Control, Cd-Cadmium, M1- *Pseudomonas aeruginosa*, M2- *Burkholderia gladioli*).

Furthermore, Cd accumulation in *L*. *esculentum* seedlings was found directly proportional to Cd concentration. The metal accumulation in roots was found to be enhanced by 505.9% in roots of Cd stressed plants as compared to control plants. Moreover, the accumulation of Cd in shoots was elevated by 109.5%. On supplementation of *P*. *aeruginosa* accumulation of Cd was reduced in roots and shoots of Cd treated seedlings by 16.43 and 13.78%. In addition, accumulation of Cd was found lowered in roots and shoots of Cd exposed seedlings by 21.83 and 7.73% by application of *B*. *gladioli* respectively (Fig. 2).

### Metal chelating compounds

An elevation in the accumulation of Cd in *Lycopersicon* seedlings triggers the synthesis of metal chelating compounds i.e protein bound thiols (PBT), non-protein bound thiols (NPBT) and total thiols. It was noticed that Cd led to significant elevation in protein bound thiols by 50.22% in Cd stressed seedlings as compared to control. With *P*. *aeruginosa* and *B*. *gladioli* application, an elevation in PBT was recorded by 77.7 and 96.2% respectively. Moreover, an increase in NBT and total thiols by 61.36 and 52.49% was recorded in Cd treated seedlings in comparison to untreated seedlings. Supplementation of both the strains in Cd exposed seedlings led to further elevation in the NBT by 36.22 and 47.45% and total thiols by 68.8 85.7% respectively (Table 2).

**Table 2:** Effect of M1 (10^9^ cells/ml) and M2 (10^9^ cells/ml) on metal chelating compounds.

Treatments	Total thiols (mmol g^−1^ FW) (Mean ± SD)	Non-protein bound thiols (mmol g^−1^ FW) (Mean ± SD)	Protein bound thiols (mmol g^−1^ FW) (Mean ± SD)
Control	0.122 ± 0.0169^e^	0.0247 ± 0.0015^d^	0.0973 ± 0.0154^d^
Cd	0.186 ± 0.0183^d^	0.0398 ± 0.0022^c^	0.1463 ± 0.0160^c^
M1	0.220 ± 0.0091^c,d^	0.0493 ± 0.0018^abc^	0.1714 ± 0.0075^c^
M1 + Cd	0.314 ± 0.0061^a^	0.0543 ± 0,0021^ab^	0.2601 ± 0.0082^a^
M2	0.252 ± 0.0123^b,c^	0.0430 ± 0.0015^bc^	0.2093 ± 0.012^b^
M2 + Cd	0.346 ± 0.0076^a^	0.0588 ± 0.0017^a^	0.2871 ± 0.0088^a^
F- ratio_(df 1,12)_ T	197.44**	2.231	160.16**
F- ratio_(df 2,12)_ D	218.11**	17.09**	178.41**
F- ratio_(df 2,12)_ T × D	2.721*	9.62**	4.35
HSD	0.0346	0.014	0.0324

^a^Total thiol content ^b^Non- protein bound thiol content and ^c^Protein bound thiol content in 10-days old *L*. *esculentum* seedlings under Cd stress (0.4 mM). Data is presented as means of 3 replicates ± S.D (standard deviation) and HSD values. F ratio values, * indicates significance at P ≤ 0.05 and ** indicates significance at P ≤ 0.01). Different letters on the graphs indicate that mean values of treatments are significantly different at p < 0.5 according to Tukey’s multiple comparison test (CN-Control, Cd-Cadmium, M1-*Pseudomonas aeruginosa*, M2- *Burkholderia gladioli*).

### Gene expression analysis

**Figure 3:**
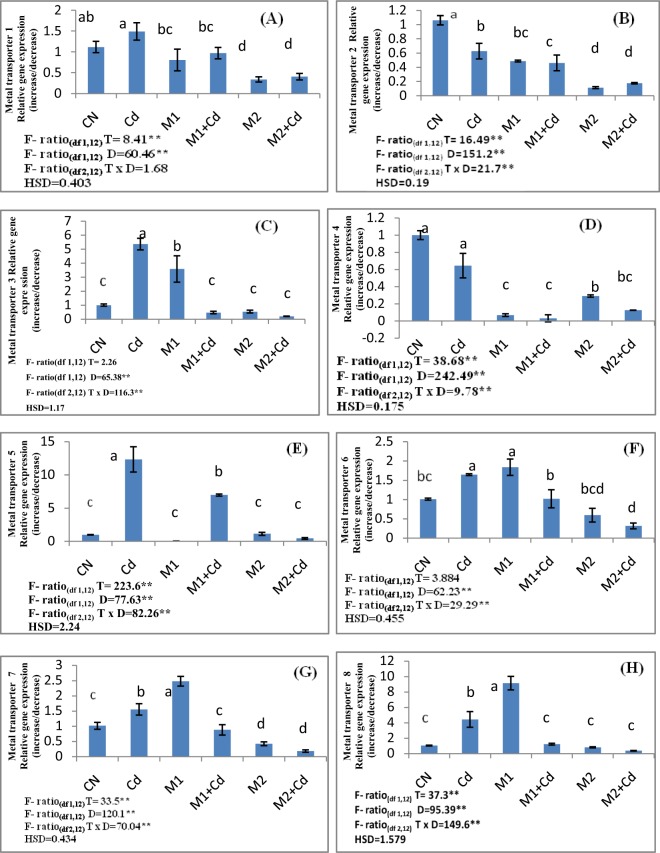
Effect of M1 (10^9^ cells/ml) and M2 (10^9^ cells/ml) on (**A**) Metal transporter1 (**B**) Metal transporter 2 (**C**) Metal transporter 3. (**D**) Metal transporter 4 (**E**) Metal transporter 5 (**F**) Metal transporter 6 (**G**) Metal transporter 7 (**H**) Metal transporter 8 in 10-days old *L*. *esculentum* seedlings under Cd stress. Data is presented as means of 3 replicates ± S.D (standard deviation) and HSD values. F ratio values, * indicates significance at P ≤ 0.05 and ** indicates significance at P ≤ 0.01). Different letters on the graphs indicate that mean values of treatments are significantly different at p < 0.5 according to Tukey’s multiple comparison test (CN-Control, Cd-Cadmium, M1-*Pseudomonas aeruginosa*, M2- *Burkholderia gladioli*).

**Figure 4:**
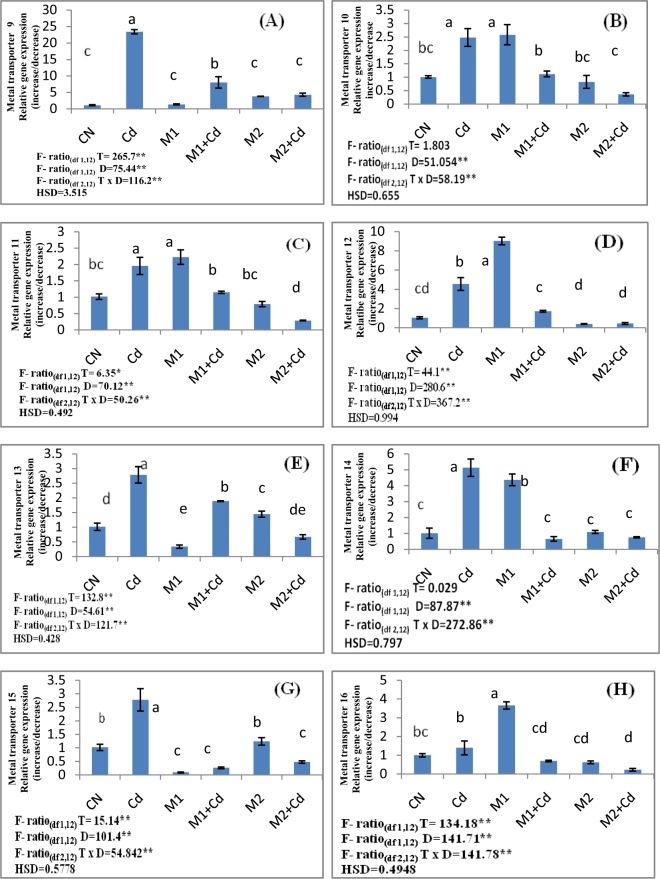
Effect of M1 (10^9^ cells/ml) and M2 (10^9^ cells/ml) on (**A**) Metal transporter 9 (**B**) Metal transporter 10 (**C**) Metal transporter 11 (**D**) Metal transporter12 (**E**) Metal transporter13 (**F**) Metal transporter14 (**G**) Metal transporter15 (**H**) Metal transporter16 in 10-days old *L*. *esculentum* seedlings under Cd stress. Data is presented as means of 3 replicates ± S.D (standard deviation) and HSD values. F ratio values, * indicates significance at P ≤ 0.05 and ** indicates significance at P ≤ 0.01). Different letters on the graphs indicate that mean values of treatments are significantly different at p < 0.5 according to Tukey’s multiple comparison test (CN-Control, Cd-Cadmium, M1-*Pseudomonas aeruginosa*, M2- *Burkholderia gladioli*).

Cd-treated *Lycopersicon* seedlings (shoots) showed a drastic increase in the gene expression of metal transporter genes under Cd stress as compared to control seedlings. Gene expression studies of metal transporter 1, metal transporter 3, metal transporter 5, metal transporter 6 and metal transporter 7 genes revealed an up regulation by 34.06, 433, 12 folds, 62.6 and 53.18% respectively. Moreover, other transporter genes i.e metal transporter 8, metal transporter 9, metal transporter 10, metal transporter 11, metal transporter 12, metal transporter 13, metal transporter 14, metal transporter 15 and metal transporter 16 showed enhanced expression in Cd treated seedlings by 320%, 23 folds, 146, 92.3, 338, 174, 404, 171 and 39.6% respectively. However, metal transporter 2 and metal transporter 4 showed a reduced expression by 41.02 and 35.56% in Cd-treated seedlings in response to control.The seedlings treated with *P*. *aeruginosa* (M1) showed decline in the expression of metal transporter 1, metal transporter 2, metal transporter 3, metal transporter 4, metal transporter 5, metal transporter 6 and metal transporter 7 by 35.04, 26.47, 91.1, 95.1, 43.4, 37.9 and 43.2% in Cd treated seedlings. However, metal transporter 8, metal transporter 9, metal transporter 10, metal transporter 11, metal transporter 12, metal transporter 13, metal transporter 14, metal transporter 15 and metal transporter 16 also showed the reduced expressions by 72.2, 65.7, 54.8, 41.2, 62.3, 32.2, 87.1, 90.4 and 50% respectively in seedlings exposed to Cd. Similar decline in the expression of metal transporter 1, metal transporter 2, metal transporter 3, metal transporter 4, metal transporter 5, metal transporter 6 and metal transporter 7 was recorded by 72.9, 72.1, 96.2, 80.5, 96.4 and 80.69% respectively on application of *B*. *gladioli* (M2) in Cd stresses seedlings. Also the expression levels of metal transporter 8, metal transporter 9, metal transporter 10, metal transporter 11, metal transporter 12, metal transporter 13, metal transporter 14, metal transporter 15 and metal transporter 16 were also lowered upon inoculation of *B*. *gladioli* (M2) in Cd treated seedlings by 88, 91.4, 85.5, 85.28, 90.1, 75, 85.1, 82.7 and 83.4% respectively (Figs 3 and 4).

## Discussion

The present study revealed that Cd metal inhibited the growth of *L*. *esculentum* seedlings in terms of root and shoot length. This decline in plant growth in the presence of Cd might be due to lowered water potential and nutrient imbalance. It can also be due to obstruction in the proton pumps that further causes impairment in the cell division and elongation^32^. Our studies are in agreement with Dutta *et al*. and Ahmad *et al*. who suggested that Cd treated *B*. *juncea* showed reduced root and shoot lengths^33,34^. Moreover, reports of decline in the root and shoot lengths due to metal toxicity have been suggested in earlier studies conducted in *Vigna mungo* exposed to Cd^33^, *Zea mays* exposed to Cu and Pb^35^, *O*. *sativa* exposed to As^36^, and *Pennisetum purpureum* exposed to Pb^37^. Suppression in the root and shoot elongation is correlated to direct inhibition of root as well as shoot metabolism^38^. Supplementation of microbial strains (*P*. *aeruginosa* and *B*. *gladioli*) significantly improved the growth of seedlings in the present study. Micro-organisms are beneficial for the plant growth and development, and helps in phosphorous uptake in plants^39^. The mechanism by which micro-organisms enable phosphate uptake is mainly through extending branching pattern and root hair formation, through hormonal stimulation. This facilitates organic phosphates via microbial turnover that induces metabolic activities involved in solubilization and mineralization of organic phosphorous. The metabolic activity involves efflux of protons and different anions followed by the release of phosphatases enzymes that lead to phosphorus hydrolysis and mineralization respectively. Stimulation of root and shoot length by microbial inoculations was also observed by Liu *et al*. in *Z*. *mays* under Cd stress^40^. It was further reported that *Enterobacter asburiae* KE17 enhanced the growth and metabolism of soybeans under Cu and Zn toxicity^41^. The increase in root and shoot length was attributed to the plant growth promoting traits possessed by the inoculated micro-organisms.

The present study revealed that Cd toxicity resulted in declined fresh weight of the seedlings. Similar reports on reduced biomass under Cd stress in *B*. *juncea* have been observed by Verma *et al*. and Dutta *et al*.^33,42^. Reduction in the plant biomass during Cd stress is mainly due to its adverse affects upon mineral uptake, photosynthesis, chlorophyll synthesis, altered water and hormonal balance^43^. Cd is first exposed to roots which later on through apoplastic pathway may enter the system and can disrupt the complete metabolism of the plant^44^. Further, fresh weight of roots and shoots were found to be lowered in Cd-treated Russian knapweed^45^. Our results revealed that inoculations of *P*. *aeruginosa* and *B*. *gladioli* improved fresh weight of *L*. *esculentum* seedlings. There is a direct correlation of plant growth and biomass with plant growth promoting rhizobacteria (PGPRs) which was found to be increased after augmentation of PGPRs under Cd toxicity^46^. An elevation in the growth characteristics was also observed in *Enterobacter* inoculated *O*. *sativa* exposed to Cd^47^. Our studies were in the agreement with the previous studies conducted by Treesubsuntorn *et al*. who found that *B*. *subtilis* and *B*. *cereus* when inoculated to Cd exposed *O*. *sativa* plants resulted in the higher root and shoot biomass^48^. This is possibly due to plant growth hormone production (IAA) by assisted microbes that regulate the hormones within plant tissues and make them acclimatize towards stress conditions^48^. Moreover, *Trichoderma* sp. elevated the plant growth characteristics such as plantbiomass, yield, germination rate and many other plant growth promoting traits under arsenic (As) toxicity^49^. It was found that As tolerant strain improved the phosphate solubilisation from soil towards plant system via phosphate and As transporters that enhanced the metabolic and physiological activities of the *Cicer arietinum* plants^49^. Several other studies in *Solanum nigrum* L.^50^, *Ocimum gratissimum L*.^51^, *Eruca sativa*^46^, *Zea mays*^52^, and *O*. *sativa*^53^ under Cd exposure showed the similar results that plant growth promoting bacteria could attribute to improved growth indices as found in our studies. Similarly, various reports of *P*. *aeruginosa* and *B*. *gladioli* in growth promotion and phosphate solubilization in heavy metal toxicity have been reported^54–56^.

The results of the present study showed that Cd toxicity reduced the contents of photosynthetic pigments such as chlorophyll, carotenoid and xanthophylls in *L*. *esculentum* seedlings. It has been observed that Cd hinders photosynthetic machinery of rice plants by altering the levels of chlorophyll a, chlorophyll b, carotenoids and net photosynthetic activity^57^. It also causes the structural changes in leaves leading to damage in their photosynthetic apparatus. It was investigated by Amirjani. that Cd exposure reduced the levels of carotenoids, total chlorophyll, chlorophyll a and chlorophyll b in *Triticum aestivum*^58^. The decline in the levels of photosynthetic pigments is mainly due to loss of cell wall and membrane integrity of thylakoid membrane. Many other heavy metals such as Zn, Fe, Cu, Hg, Cr and Pb leads to impediment of enzymes such as rubisco, chlorophyll synthase, protochlorophyllide reductase and δ-aminolevulinic acid dehydratase involved in the synthesis of chlorophyll^59^, that result in the breakdown of chlorophyll pigments^60^. Moreover, Cd- mediated breakdown of chlorophyll might be due to the activation of enzymes involved in hydrolysis of chlorophyll such as chlorophyllase^61^. They also lead to the activation of xanthophyll cycle in order to protect the photosynthetic apparatus from the metal exposure^62^. Moreover, carotenoids protect the plant’s photosynthetic machinery from photo-oxidative disruptions through ROS scavenging. Reduction in carotenoids lead to PSII damage by retrogression of D1 protein that inhibits chlorophyll synthesis^63^. The observations made in our study are in accordance with Chen *et al*. who reported decline in the chlorophyll and carotenoid levels in Cd treated *B*. *campestris* and *B*. *juncea*^44^. Enhancement in the photosynthetic pigments could occur in the presence of plant growth promoting bacteria that increases nutrient uptake in plants through phosphate solubilization and exudating essential substances that play crucial role in synthesis of photosynthetic pigments required for light harvesting complex and its photo assimilation^64^. Inoculation of *Klebsiella pneumoniae* in *V*. *mungo* enhanced the levels of chlorophyll under Cd stress^33^. It was suggested by Rizvi *et al*. that *Azotobacter chrococcum* when supplemented with Cu and Pb exposed *Zea mays* plants enhanced the chlorophyll contents^35^.

Furthermore, elevation in chlorophyll as well as carotenoid contents in *O*. *sativa* was observed upon inoculations with *Pseudomonas putida* and *C*. *vulgaris* upon As treatment which is attributed to lessened accumulation of As that prevented the toxicity symptoms of As. We can speculate that it is most likely due to the growth promoting effects of PGPRs as well as enhanced protein levels that in turn stimulated the pigment levels. Several studies of PGPRs in enhancing the levels of chlorophyll and carotenoids under Cd stress have been reported such as *Proteus vulgaris* in pigeon pea^65^, *Enterobacter* in rice^47^, *Azotobacter* in *Plantago ovata*^66^, and *K*. *pneumoniae* in rice^53^.

In the current study, Cd stress lowered the heavy metal tolerance index and enhanced Cd- uptake in *L*. *esculentum* seedlings. It was demonstrated that inhibition of seed germination in various cereal crops such as rice, wheat and barley in response to different heavy metals is probably due to the morphological and physiological changes in roots that results in reducing heavy metal tolerance^67^. The results also signifies that treatment of seedlings with microbial strains (*P*. *aeruginosa* and *B*. *gladioli*) individually mitigates Cd induced reduction in heavy metal tolerance index. Heavy metal tolerance index in Ni and Cd treated tomato seedlings was reduced upon increased concentrations of Ni and Cd which upon supplementation of *Methylobacterium oryzae* and *Burkholderia* sp. was enhanced^68^. According to them, these metals raised stress marker ethylene levels which was reduced upon microbial inoculations. Along with this, they also observed that microbes precipitated heavy metals and lowered their availability by binding to functional groups such as hydroxyl, carboxyl, amide group etc. and chelating them via extracellular produced polymers such as humic substances and polysaccarides^68^. The results of the present study showed that Cd content was more in roots as compared to shoots. Similarly, studies with enhanced accumulation of different metal ions in roots were reported in *Z*. *mays* under Cu stress^35^, *O*. *sativa* under As stress^36^, and rapeseed under Cd presence^69^. The reduction in the uptake and translocation of heavy metals towards shoots is the adaptive measure adopted by the plants under stressful conditions^70^. Augmentation of micro-organisms in the present study reduced the Cd accumulation in roots as well as shoots. Previous studies conducted by Rizvi *et al*. suggested that *Z*. *mays* when inoculated with *A*. *chroococcum* reduced the Cu and Pb accumulation in plant organs which is most likely due to the secretion of different metabolites, protons and exudates that act as metal chelators and immobilise Pb^35^. Moreover, it was reported that metal tolerant strain *Bacillus megaterium* reduced the Ni translocation^20^, and As-tolerant *Exiguobacterium* reduced As translocation in *Vigna radiata* plants by colonization at the root surfaces^71^. It has been found that *Acinetobacter lwofii* promotes the growth and reduces As uptake in *V*. *radiata*^72^ They speculated that *A*. *lwofii* produced growth promoting substances such as indole-3 acetic acid and siderophores that help them to resist the metal contamination. In addition, they also form biofilms which restricted the As uptake in these plants^72^.

Molecular studies revealed that PGPRs led to As biotransformation in wheat plants where up regulation of *arsC*, *aioA* and *arsM* genes reduced As levels in roots as well as shoots^10,73^. Furthermore, metal homeostasis within plants is mainly regulated by metal transporters and transport proteins depending upon the bioavailability of these metals^74^. In present study, gene expression profiling of metal transporter genes in seedlings under Cd metal stress have been studied through qRT-PCR. It was observed that expression of metal transporters were enhanced that resulted in the accumulation of Cd in roots and shoots which were lowered in the presence of microbes. A study conducted by Chen *et al*. showed that cloning of As- antiporter *ACR3 Pteris vittata*, *PvACR3;*1 was done and expressed in *Arabidopsis thaliana* and *Nicotiana tabacum* and yeast^75^. The results observed by them in yeast indicated that *PvACR3;*1 is a well developed antiporter that efflux As into the medium. Moreover, they also found that *Arabidopsis thaliana* and *Nicotiana tabacum* accumulated higher levels of As and reduced their accumulation in shoots^75^.

It was further reported that *Pseudomonas putida* possesses very well developed CzcCBA efflux system which involved three main genes *CzcA*, *CzcB* and *CzcC* and effect of these genes were studied in tobacco plants under Cd exposure^76^. They postulated that *CzcB* and *CzcC* genes led to lowered accumulation of Cd in shoots thereby reducing the Cd toxicity in contaminated soils. Similar studies revealed by Manzoor *et al*.depicted that microbes such as *Pseudomonas* possess metal resistance genes such as *CzcR*, *Pbr A*, *CadA2* and *ZntA* that encodes different efflux proteins involved in metal detoxification processess. These genes belongs to P_IB_- type ATPases that transport metal ions from cytoplasm towards periplasm and precipitated to prevent the entry into the nearby cells^77^. Moreover, *CzcA* gene (encoded by bacteria) is a key antiporter and it has been found to be involved in controlling metal ion bioavailability at contaminated sites^78^. Therefore, the reduction of metal uptake via inoculation of plant growth promoting microbes enabled the *Lycopersicon* plants to survive better under metal exposure by detoxifying them that would otherwise affected the overall plant health.

The present study also revealed that metal chelating compounds such as total thiols, protein bound thiols and non-protein bound thiols were enhanced in seedlings under Cd stress. These metal chelators usually consist of sulfhydrl (-SH) groups that effectively bind to metals in order to immobilise them^1^. Our studies are in accordance with the studies of Aly and Mohamed. in *Scenedemus bijugatus* and maize under Cu stress^79^. Moreover, Cu accumulation can modulate glutathione levels which further stimulates the metal binding properties and sequester the metals^80^. It has been studied that thiol metabolism is the most crucial pathway in regulation of heavy metal stress tolerance. It mainly consists of non-protein thiols (NBTs), phytochelatins, protein thiols and glutathione that form complexes with metals and mediate their transport and sequestration into the vacuoles^81^. Moreover, NBT imparts antioxidant properties to the plants. The most important metabolite of thiol metabolism, cysteine produced during sulphur assimilation leads to the synthesis of phytochelatins and glutathione. The supplementation of *P*. *putida* in rice plants under As toxicity enhanced the levels of NBTs, phytochelatins and glutathione. According to their study, it is most probably due to complexation of As in shoots and roots^36^. This elevation in the levels of thiols might be due to increased nutrient uptake of different nutrients such as nitrogen, phosphorous, magnesium, potassium and sulphur in the presence of micro-organisms that stimulate the synthesis of these metal chelating compounds.

## Conclusions

The results of the present study concluded that presence of plant growth promoting rhizobacterial strains *P*. *aeruginosa* and *B*. *gladioli* in soils promoted growth of plants in terms of root length, shoot length and fresh weight in *L*. *esculentum* seedlings subjected to Cd stress. A significant increase in the photosynthetic efficiency was also observed in Cd exposed microbe inoculated seedlings by elevating the contents of photosynthetic pigments such as chlorophyll, carotenoid and xanthophylls. Further, it was found that presence of these microbes reduced the Cd metal accumulation in the seedlings. The decreased levels of Cd resulted in alleviation of Cd toxicity through decreasing its bioavailability. The effective role played by microbes in reduction of metal uptake is mainly due to immobilization that results in binding the metal to root by complex formation which further prevent its translocation towards shoot. The role of metal chelating compounds such as non-protein thiols and protein thiols have also been elucidated in binding actions of heavy metals by microbes. Moreover, PGPRs also promote plant growth by enhancing mineral uptake, phytohormone production and nitrogen fixation. Therefore, all these traits act as driving force in enhancing the growth of plants under metal polluted environment. The present study therefore projects the contribution of micro-organisms in decreasing Cd toxicity and accumulation, implicating their roles for achieving the goal of lower Cd concentrations in tomato plants with better growth conditions.

## Materials and Methods

### Inoculation of microbial strains

Selected microbial strains (*Pseudomonas aeruginosa* strain no. MTCC7195) and *Burkholderia gladioli* strain no. MTCC10242) were obtained from IMTECH, Mohali, Punjab (India). These strains were cultured individually by addition of bacterial strains in 50 mL of nutrient broth (13 gL^−1^) medium. The flasks were later placed in the BOD incubator (Caltan (Deluxe Automatic), New Delhi, India) for 24–48 h at 28 °C for their proliferation. After their growth sub-culturing was done so as to maintain them for future. The culture was grown by adding 1 mL of above grown pure culture of bacteria (10^9^ cells/mL) in 50 mL nutrient broth at 28 °C for 24–48 hrs. It was then centrifuged at 8000 rpm, 4 °C for 15 minutes to obtain the pellet. Pellet was washed twice using distilled water and resuspended in distilled water. The concentration of 10^9^ cells/mL was adjusted for the experimental purpose.

### Plant material and treatments

The certified seeds of *L*. *esculentum* (tomato) var. Pusa Ruby were sterilized by using 0.01% mercuric chloride (HgCl_2_) solution. The seeds were dipped for a minute and rinsed thoroughly for 5–6 times using distilled water. Petriplates were lined with Whatman filter paper (grade 1 with diameter 10 cm) onto which thirty seeds were raised by soaking the seeds with microbial suspensions (having diameter approx. 10 cm) at 10^9^ cells/mL concentration and CdCl_2_ solution containing Cd (0.4 mM). The Petri plates were tracked in seed germinator for 10 days at controlled conditions of light (16-h photoperiod with white fluorescent light intensity 175 μmol m^−2^ s^−1^), temperature (22–25 °C), and relative humidity (80–90%) in seed germinator. Cd concentration was selected on the basis of IC50 (0.4 mM) value.

### Growth Parameters

Growth parameters were assessed after harvesting the seedlings by examining their root length and shoot length. Fresh weight was also recorded from the harvested seedlings after 10-days.

### Evaluation of Heavy metal tolerance index

Heavy metal tolerance index was calculated using method of Balint *et al*.^82^. For this, dry weights of seedling samples were taken and tolerance index was calculated using the formula given below:

$$ \% \,{\rm{Heavy}}\,{\rm{metal}}\,{\rm{tolerance}}\,{\rm{index}}=\tfrac{{\rm{Dry}}\,{\rm{weight}}\,{\rm{of}}\,{\rm{Treated}}\,{\rm{Plants}}}{{\rm{Dry}}\,{\rm{weights}}\,{\rm{of}}\,{\rm{Untreated}}\,{\rm{plants}}\,(\mathrm{CN})}\times 100$$

### Metal accumulation

Cd- metal accumulation in plant tissues (roots and shoots) was estimated by using Atomic Absorption Spectrophotometer (Shimadzu 6200, Agilent technologies GTA 120). The seedlings were harvested and their roots and shoots were separated and allowed to dry in oven at 65 °C for 48 hrs. The dried plant samples were further digested using the method proposed by Allen *et al*.^83^. For digestion, 200 mg of powdered sample was taken which was then digested in aqua regia (H_2_SO_4_: HNO_3_: HClO_4_, v/v) in ratio 1:3:1 using glass beakers on a hot induction plate. After that, digested samples were cooled and filtered using 0.22-μm nylon syringe filters. The samples were further diluted using double distilled water to make up the final volume upto 50 ml. These digested samples were stored at room temperature and used for further analysis.

### Photosynthetic pigments

#### Determination of chlorophyll and carotenoid content

Estimation of chlorophyll and carotenoid content was done by the method given by Arnon^84^ and Maclachlan and Zalik^85^, respectively. For this purpose, 1 g of fresh seedlings were macerated in 4 mL of 80% acetone. It was further centrifuged at 12,000 rpm for 15–20 min at 4 °C. The supernatant was taken for estimation of chlorophyll and carotenoid contents. Absorbance at 645 nm and 663 nm was taken for chlorophyll analysis, whereas absorbance at 480 and 510 nm was measured for carotenoids using spectrophotometer (Thermo electron corporation, Genesys 10 UV).

#### Determination of xanthophyll content

Estimation of xanthophyll content was done by method proposed by Lawrence^86^. For this purpose, 50 mg of oven dried powdered seedling sample was kept in a 100-mL volumetric flask. To this, addition of 30 mL of combined extract (hexane (10 mL):acetone (7 mL):absolute alcohol (6 mL):toluene (7 mL)) was done and flask was shaken for 15–20 min. It was then followed by mixing 40% methanolic KOH (2 mL) to the flask. The flask was then kept in the water bath (58 °C) for 20–25 min after which the samples were placed under dark conditions for an hour. 30 mL of hexane along with 10% sodium sulfate was mixed to a volume of 100 mL followed by vigorous shaking for a minute. Again the flask was incubated under dark conditions. The upper layer was shifted into a 50-mL volumetric flask and volume was adjusted using hexane. Absorbance was taken at 474 nm.

### Metal chelating compounds

#### Total thiols

Determination of total thiols was done by homogenising 0.5 g of seedling sample in 20 mM ascorbate buffer prepared using 20 mM EDTA (Ethylene diamine tetra acetic acid). Centrifugation was carried out at 12,000 g for 15–20 min, at 4 °C. To 0.5 mL of above obtained supernatant 2.4 mL of 200 mM Tris HCl and 10 mM of DTNB (5,5-dithio-bis-[2-nitrobenzoic acid]) was mixed. It was then allowed to stand for 20 min after which absorbance was recorded at 412 nm^87^.

#### Non-protein thiols

0.5 g of fresh seedlings were macerated in 3 mL of ice cold 5% sulfosalicylic acid. The sample was then centrifuged at 12,000 g for 15–20 min at 4 °C. To 100 µL of extract, PPB (potassium phosphate buffer, 0.1 M; pH-7), 0.5 mL of 1 mM DTNB and 0.5 M EDTA was mixed. It was then placed for 15 minutes and absorbance was noted at 412 nm^88^.

#### Protein bound thiols

Estimation of protein bound thiols was done by subtraction of the non protein thiols (NPT) from total thiols (TT).

#### Gene expression analysis of metal transporters

RNA was isolated from tomato seedlings using Trizol method (Invitrogen, Life Technologies, USA). Analysis of RNA was done qualitatively as well as quantitatively through agarose gel electrophoresis (2%) and Nano Drop spectrophotometer (By Thermo Scientific, USA). The RNA isolated was treated with DNase (DNA-free TM kit; Ambion TURBO DNA-freeTM, From Life Technologies, USA) in order to restrict DNA contamination. Further, cDNA was synthesised with ImProm-IITM Reverse Transcription System (Promega, Madison, USA) using 1 *μ*g of RNA (treated by DNase) as template and oligodT12 primer (First Choice RLM-RACE Kit, Ambion, Life Technologies, Carlsbad, USA)^89^. The primers used in the current study are listed in the Table 3 (Integrated DNA technologies, USA).

**Table 3:** Nucleotide sequences used in experiment.

Metal transporter genes	Primer code	Primer Sequences	T_m_ (·C)
SGN-U143555 *At5g21930*; ATPase, E1-E2 type transporter family (Metal transporter 1)	SGN-U14F26 SGN-U14R26	Forward primer GCTTCAAATGCAGCATCC Reverse primer TCCAGCTGCAATGGGTA	60.3 59.7
SGN-U165841 *At5g21930*.*1*; ATPase, E1-E2 type transporter family (Metal transporter 2)	SGN-U16F27 SGN-U16R27	Forward primer GCTTTATTAGGACCTGGACG Reverse primer TCCCATTGAAGCTCAGAGTTA	60.1 60.0
SGN-U166596 *At4g33525*.*1;* Metal transporting P-Type ATPase (Metal transporter 3)	SGN-U16F28 SGN-U16R28	Forward primer TTCTAGCAGTCTGTCAGTCG Reverse primer TTCTAGCAGTCTGTCAGTCG	59.9 60.2
SGN-U213018 *At5g21930*.*1;* Metal transporting ATPase like protein (Metal transporter 4)	SGN-U21F29 SGN-U21R29	Forward primer GCTTCAAATGCAGCATCC Reverse primer TCCAGCTGCAATGGGTA	60.3 59.7
SGN-U236090 *At5g21930;* PAA2 (P- type ATPase metal transporter family) (Metal transporter 5)	SGN-U23F30 SGN-U23R30	Forward primer GCTTTATTAGGACCTGGACG Reverse primer TCCCATTGAAGCTCAGAGTTA	60.1 60.0
SGN-U239322 *At4g33520*.*3;* PAA1 (P- type ATPase 1 metal transporter family) (Metal transporter 6)	SGN-U23F31 SGN-U23R31	Forward primer TTCTAGCAGTCTGTCAGTCG Reverse primer AGGTTTATACTTCCTGCGG	59.9 60.2
SGN-U318095 *At5G21930*.*2;* PAA2 (P- type ATPase metal transporter family) (Metal transporter 7)	SGN-U31F32 SGN-U31R32	Forward primer GCTTCAAATGCAGCATCC Reverse primer TCCAGCTGCAATGGGTA	60.3 59.7
SGN-U320813 *At5G21930*.*1;* PAA2 (P- type ATPase metal transporter family) (Metal transporter 8)	SGN-U32F33 SGN-U32R33	Forward primer TAATCGAGTGGCTTTCGC Reverse primer GTCTCGTCCAGGTCCTAA	59.9 60.0
SGN-U321017 *At4g33520*.*2;* HMA6, PAA1 (Metal transporting P-type ATPase 1) (Metal transporter 9)	SGN-U32F34 SGN-U32R34	Forward primer TAGCAATTGGTGGTGGAGT Reverse primer TGTTGTATCCGAAGGCCC	60.4 60.3
SGN-U342455 *At4G33520*.*2;* HMA6, PAA1 (Metal transporting P-type ATPase 1) (Metal transporter 10)	SGN-U34F35 SGN-U34R35	Forward primer TTCTAGCAGTCTGTCAGTCG Reverse primer AGGTTTATACTTCCTGCGG	59.9 60.2
SGN-U574793 *At5g21930*.*1*; ATPase, E1-E2 type transporter family (Metal transporter 11)	SGN-U5F36 SGN-U5R36	Forward primer TTCTAGCAGTCTGTCAGTCG Reverse primer AGGTTTATACTTCCTGCGG	59.9 60.2
SGN-U584889 *At5g21930;* ATPase, E1-E2 type transporter family (Metal transporter 12)	SGN-U5F37 SGN-U5R37	Forward primer TAATCGAGTGGCTTTCGC Reverse primer GTCTCGTCCAGGTCCTAA	59.9 60.0
SGN-U585274 *At5g21930;* ATPase, E1-E2 type transporter family (Metal transporter 13)	SGN-U58F38 SGN-U58R38	Forward primer TAGCAATTGGTGGTGGAGT Reverse primer TGTTGTATCCGAAGGCCC	60.4 60.3
SGN-U527536 *At5g21930;* ATPase, E1-E2 type transporter family (Metal transporter 14)	SGN-U52F39 SGN-U52R39	Forward primer TTCTAGCAGTCTGTCAGTCG Reverse primer AGGTTTATACTTCCTGCGG	59.9 60.2
SGN-U535703 *At5g21930;* ATPase, E1-E2 type transporter family (Metal transporter 15)	SGN-U53F40 SGN-U53R40	Forward primer TAGCAATTGGTGGTGGAGT Reverse primer TGTTGTATCCGAAGGCCC	60.4 60.3
SGN-U542816 *At5g21930;* ATPase, E1-E2 type transporter family (Metal transporter 16)	SGN-U54F41 SGN-U54R41	Forward primer TAATCGAGTGGCTTTCGC Reverse primer GTCTCGTCCAGGTCCTAA	59.9 60.0

#### Gene expression studies using qRT-PCR

Molecular studies in 10-day old tomato seedlings subjected to different treatments were done by expression studies of different metal transporter genes mentioned in Table 1. using quantitative real time RT-PCR (qRT-PCR)^90^. IM- Prom-IITM Reverse Transcription System (Promega, USA) was used for cDNA preparation. The primers designed for qRT-PCR profiling was carried out using Primer3 software. Expression study was preceded according to the manual instructions given along with the instrument Light Cycler 96 Real Time PCR System (Hoffmann-La Roche, Switzerland). PCR reaction (20 *μ*L) preparation was done by combining the following reaction mixtures: cDNA (appropriately diluted), 1× Light Cycler 480 SYBR Green I Master (Hoffmann-La Roche, Switzerland) and 1 *μ*M primers (Integrated DNA Technologies, USA, refer Table 1 for sequences). The prerequisite for qRT-PCR (thermal cycler) were: incubation period at 95 °C for 10–15 min, followed by 45 cycles of 3 step amplification (95 °C–10 s, 60 °C–15 s and 72 °C–25 s). Data interpretation was done through dissociation curve (heating upto 95 °C–10 s under normal conditions and cooling to 65 °C–60 s). Later, heat was given slowly upto 97 °C (1 s) at lowered ramping rate of 0.2 °C/s to check distinct qPCR reaction rate. Data was assessed in triplicates that also includes the negative control (non-template). *Ubq* (*Ubiquitin*) gene was used as house-keeping control gene for normalization purposes. The data was calculated using threshold cycle (*Ct*) of the amplification curve. The relative gene expression level was assessed using the 2^−ΔΔ^^ct^ method^91,92^ (where *Ct* = (*Ct*, *target* − *Ct*, *Ubiquitin*)*time* × − (*Ct*, *target* −*Ct*, *Ubiquitin time* 0).

### Statistical Analysis

The results of the present study were interpreted statistically through two- way analysis of variance (ANOVA) and Tukey’s multiple comparison test to find the HSD (Honestly Significant Difference) among means. The values are mentioned as means ± standard deviation (S.D) and significant differences were checked at p ≤ 0.05 and 0.01. The data was examined in well built software in Microsoft excel.
